# Increased, Durable B-Cell and ADCC Responses Associated with T-Helper Cell Responses to HIV-1 Envelope in Macaques Vaccinated with gp140 Occluded at the CD4 Receptor Binding Site

**DOI:** 10.1128/JVI.00811-17

**Published:** 2017-09-12

**Authors:** Willy M. J. M. Bogers, Susan W. Barnett, Herman Oostermeijer, Ivonne G. Nieuwenhuis, Niels Beenhakker, Daniella Mortier, Petra Mooij, Gerrit Koopman, Edmund Remarque, Gregoire Martin, Rachel Pei-Jen Lai, Antu K. Dey, Yide Sun, Brian Burke, Guido Ferrari, David Montefiori, Loic Martin, David Davis, Indresh Srivastava, Jonathan L. Heeney

**Affiliations:** a Department of Virology, Biomedical Primate Research Centre (BPRC), Rijswijk, The Netherlands; b CEA, Joliot, Service d'Ingénierie Moléculaire des Protéines, Gif sur Yvette, France; c Laboratory of Viral Zoonotics, Department of Veterinary Medicine, University of Cambridge, Cambridge, United Kingdom; d Novartis Vaccines and Diagnostics Corporation, Emeryville, California, USA; e Duke University Medical Center, Durham, North Carolina, USA; Emory University

**Keywords:** B-cell responses, CD4 mimetic, CD4 occluded, nonhuman primates, T-cell responses, human immunodeficiency virus, vaccines

## Abstract

Strategies are needed to improve the immunogenicity of HIV-1 envelope (Env) antigens (Ag) for more long-lived, efficacious HIV-1 vaccine-induced B-cell responses. HIV-1 Env gp140 (native or uncleaved molecules) or gp120 monomeric proteins elicit relatively poor B-cell responses which are short-lived. We hypothesized that Env engagement of the CD4 receptor on T-helper cells results in anergic effects on T-cell recruitment and consequently a lack of strong, robust, and durable B-memory responses. To test this hypothesis, we occluded the CD4 binding site (CD4bs) of gp140 by stable cross-linking with a 3-kDa CD4 miniprotein mimetic, serving to block ligation of gp140 on CD4^+^ T cells while preserving CD4-inducible (CDi) neutralizing epitopes targeted by antibody-dependent cellular cytotoxicity (ADCC) effector responses. Importantly, immunization of rhesus macaques consistently gave superior B-cell (*P* < 0.001) response kinetics and superior ADCC (*P* < 0.014) in a group receiving the CD4bs-occluded vaccine compared to those of animals immunized with gp140. Of the cytokines examined, Ag-specific interleukin-4 (IL-4) T-helper enzyme-linked immunosorbent spot (ELISpot) assays of the CD4bs-occluded group increased earlier (*P* = 0.025) during the inductive phase. Importantly, CD4bs-occluded gp140 antigen induced superior B-cell and ADCC responses, and the elevated B-cell responses proved to be remarkably durable, lasting more than 60 weeks postimmunization.

**IMPORTANCE** Attempts to develop HIV vaccines capable of inducing potent and durable B-cell responses have been unsuccessful until now. Antigen-specific B-cell development and affinity maturation occurs in germinal centers in lymphoid follicles through a critical interaction between B cells and T follicular helper cells. The HIV envelope binds the CD4 receptor on T cells as soluble shed antigen or as antigen-antibody complexes, causing impairment in the activation of these specialized CD4-positive T cells. We proposed that CD4-binding impairment is partly responsible for the relatively poor B-cell responses to HIV envelope-based vaccines. To test this hypothesis, we blocked the CD4 binding site of the envelope antigen and compared it to currently used unblocked envelope protein. We found superior and durable B-cell responses in macaques vaccinated with an occluded CD4 binding site on the HIV envelope antigen, demonstrating a potentially important new direction in future design of new HIV vaccines.

## INTRODUCTION

Antibody (Ab) responses directed to the human immunodeficiency type 1 (HIV-1) envelope have been correlated with protection from viral infection; however, the ability to induce the B-cell responses necessary to generate long-lived protective antibodies by vaccination has proven difficult. Impressive protection from *in vivo* challenge has been achieved repeatedly using passive transfer of broadly neutralizing monoclonal antibodies (bNAb), and these approaches now are being advanced by multiple groups to clinical proof of concept. Such bNAbs have been cloned from memory B cells from HIV-1-infected patients, and sequence analysis has revealed substantial somatic hypermutation (SHM) from the parental Ig germ line (1) characteristic of high-affinity maturation of antigen-specific B cells in germinal centers. CD4-positive T follicular helper cells (Tfh) play a fundamental role in Ab maturation by promoting Ig class switch recombination (CSR), SHM, B-cell selection, and differentiation. A deeper understanding of these events may provide insights for improved HIV vaccine design.

The close interaction of activated Ag-specific CD4 T cells and major histocompatibility complex class II (MHC-II) B cells within germinal centers is critical for optimal development of anti-HIV Ab responses. Priming of naive CD4^+^ T cells is initiated by MHC-II-positive dendritic cells in lymph nodes to differentiate into Tfh cells prior to their migration to the T-cell–B-cell interfaces of germinal centers (GC) (2–4). This promotes their encounter with B cells that share the same Ag specificity, reinforcing their lineage commitment and coalescence, mutual activation, and formation of GC. Importantly, it is the intensity of the Tfh signal which is dictated by the quality and longevity of B-cell interactions with molecules expressed on the surface of Tfh cells (5). Signals from Tfh are critical for differentiation of GC B cells into memory B cells and long-lived plasma cells and their maturation of Ig affinity by CSR and SHM (6). The B cells with the strongest Tfh cell interactions are those that become memory B cells or leave the GCs and differentiate into long-lived plasma cells (7). Importantly, the cytokines interleukin-21 (IL-21), IL-6, and IL-4 play key roles in affinity maturation of Igs in B cells. IL-21, which is central to Tfh development, is augmented by IL-4, and together they collaborate to promote Ig responses (8). Additionally, the tight regulatory program between Ag-specific B cells in germinal centers is enhanced by the circulation and exchange of Tfh cells between B-cell-rich germinal centers to ensure maximal diversification of CD4 T-cell help.

Factors which interfere with Tfh activation and collaboration with B-cell development have a negative response on maturation of Ig responses and ultimately on their effector function. Of the viruses which cause persistent infection, HIV is unique in that it utilizes the CD4 receptor with a specific high-affinity CD4 binding site (CD4bs) on the envelope subunit gp120. Through the CD4bs, the HIV Env gp120 subunit can bind to the CD4 receptor in the absence of an intact infectious virion, either as a monomer or in its trimeric form (gp120 or gp140). Notably, HIV-1, HIV-2, and simian immunodeficiency viruses (SIVs) infect Tfh cells in GCs (9), and the Tfh population serves as the major T-cell compartment for HIV infection, replication, and production (10), ultimately contributing to the loss of CD4 T cells and immune deficiency. However, very early in infection, before numerical CD4 T-cell loss, HIV causes defects in CD4 T-cell and MHC-II antigen-presenting cell (APC) function, defects which also affect B-cell responses to infection (11, 12).

A growing number of studies have confirmed that gp120 alone or immune complexed with antibodies is likely to decrease CD4 T-cell function (13). HIV-1 replication is associated not only with virion-bound Env glycoprotein but also with shedding of soluble gp120 or gp160 during replication *in vivo* (14, 15). Soluble gp120 is found in plasma (16–18) and lymphoid tissues of HIV^+^ patients (19, 20). This has been found to correlate with dysfunctional CD4^+^ T-cell responses (18, 21, 22). A number of studies have demonstrated that gp120 binding to the CD4 receptor interferes with normal T-cell receptor (TCR)-induced CD4^+^ T cell activation (19–21). The recent confirmation that gp120-immune complexes also engaged CD4 receptors and prevented subsequent TCR-mediated activation of CD4^+^ T cells has raised concerns over immunization with HIV envelope (13).

Here, we set out to examine the hypothesis that in a vaccine setting, CD4 binding HIV-1 envelope immunogens could be detrimental to achieving optimal vaccine-induced B-cell responses. To study this, we compared an HIV-1 gp140 immunogen in which the binding to CD4 on the surface of T cells was abrogated by stable complexing of the antigen with a CD4 miniprotein mimetic (termed mini-CD4) that served to occlude the CD4bs on the Env molecule. For this purpose, as a scaffold molecule we used scyllatoxin, from the scorpion Leiurus quinquestriatus, which mimics features of the CD4 receptor that actually binds to the HIV Env glycoprotein (the β-hairpin of scyllatoxin can be superimposed onto positions 36 to 47 of the CDR2 loop of the CD4 molecule). Subsequent transfer of the side chains of the amino acids of CD4 to the equivalent positions of scyllatoxin resulted in a minipeptide which specifically binds to the HIV-1 Env glycoprotein CD4bs with affinity for HIV-1 equivalent to that of CD4 itself (22). Notably, structural data demonstrate that the M64U1 mimetic occludes the CD4 S-375 HIV-1 protein residue (23), which has been reported to enhance HIV-1 Env CD4 binding and virus replication in macaques (24). Importantly, our design provided both CD4bs occlusion, blocking high-affinity binding Env residues, such as S-375, while allowing preservation of CD4-inducible (CD4i) neutralizing antibody epitopes (with putative antibody-dependent cellular cytotoxicity [ADCC] epitopes), both of which we have demonstrated in previous structural and small-animal studies (22, 25, 26).

Since nonhuman primates have CD4 receptors on T cells that are functionally and structurally very similar to human CD4, we studied these receptors using 4 groups with a total of 24 macaques to determine if vaccine-induced B-cell responses and effector responses, such as ADCC, could be improved by preventing CD4 engagement. Furthermore, to determine if the effect was determined at the level of CD4 T cells, we enumerated antigen-specific CD4 T cell subsets that secreted gamma interferon (IFN-γ), IL-2, or IL-4 to determine which of these subsets correlates with the observed B-cell and/or ADCC responses.

## RESULTS

### Immunization kinetics of B-cell responses in rhesus macaques immunized with HIV-1 Env occluded at the CD4 binding site.

**FIG 1:**
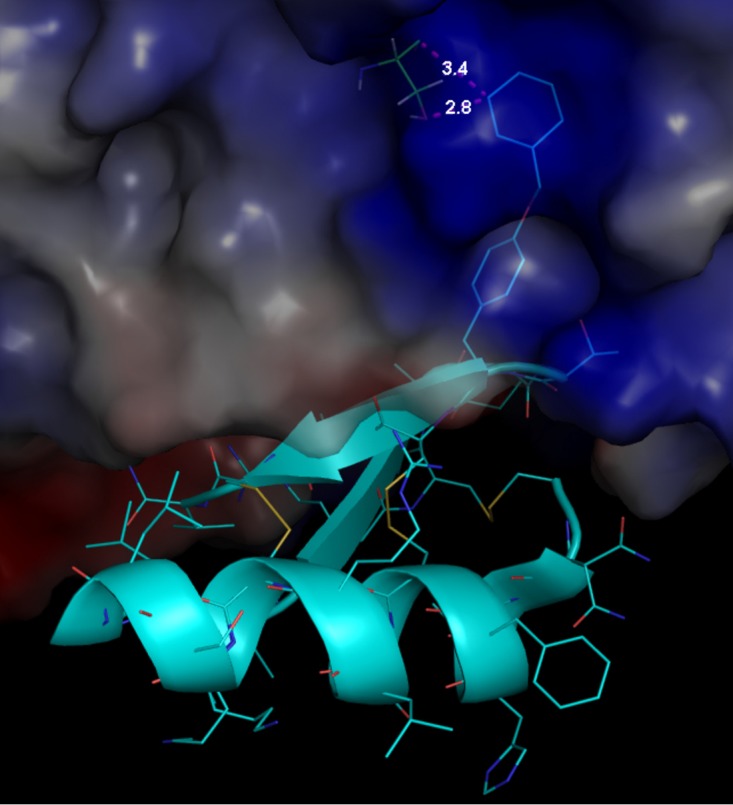
Zoomed-in view of M48U1 binding site showing close contact (pink dotted line) between M48U1 cyclohexylmethoxy group Phe23 and gp120YU2 Ser375. gp120 is shown as a transparent surface. M48U1 is shown in cyan cartoon representation with its 23 residues represented as sticks. The illustration was prepared with PyMOL 1.8.2.1 using PDB entry 4JZZ. The interaction between cyclohexylmethoxy (U1) in M48U1 with both main-chain and side-chain O atoms of Ser375 gp120 (23) is shown.

**FIG 2:**
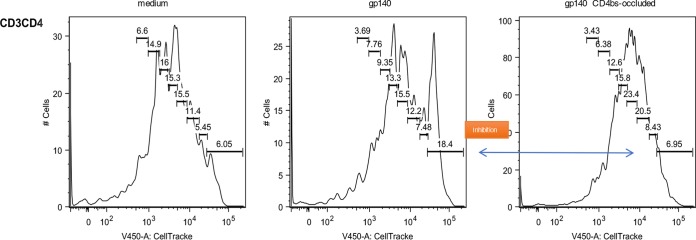
SF162 gp140-mediated inhibition of CD3/TCR-induced CD4 T-cell proliferation is prevented by CD4bs occlusion. In each graph, bars are used to indicate the number of undivided cells (bar on the right) and the number of cells for each cell division within the CD3CD4 population. Note the difference in the fraction of undivided cells, which is increased after addition of SF162 gp140 (CD4bs-open) but not of SF162 gp140 (CD4bs-occluded).

**FIG 3:**
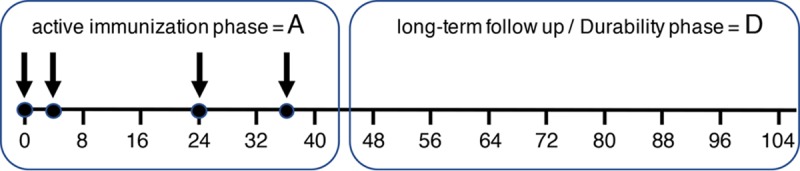
Immunization schedule and long-term (17.5 months/70 weeks) follow-up depicting the immunization phase (box A) and durability phase (box D).

We set out to test our hypothesis that CD4 binding HIV-1 envelope immunogens are detrimental to achieving optimal and durable vaccine-induced B-cell responses. In addition to examining the structural evidence of occlusion the CD4bs on the Env protein (Fig. 1) (23), we performed an exploratory fluorescence-activated cell sorting (FACS)-based assay to confirm the inhibitory effect of SF162 gp140 on rhesus CD4 T-cell proliferation in the presence of anti-CD3 *in vitro* (Fig. 2). This assay confirmed that gp140 inhibition of CD4 T-cell proliferation was abrogated by using the M48U1 CD4bs-occluded form of Env protein. The vaccine study was performed in 24 rhesus macaques divided into the immunization-active and long-term follow-up or durability phase, as indicated in Fig. 3. Animals were immunized four times over 40 weeks (Fig. 3, phase A), and vaccine-induced antigen-specific B cells in peripheral blood were enumerated.

**FIG 4:**
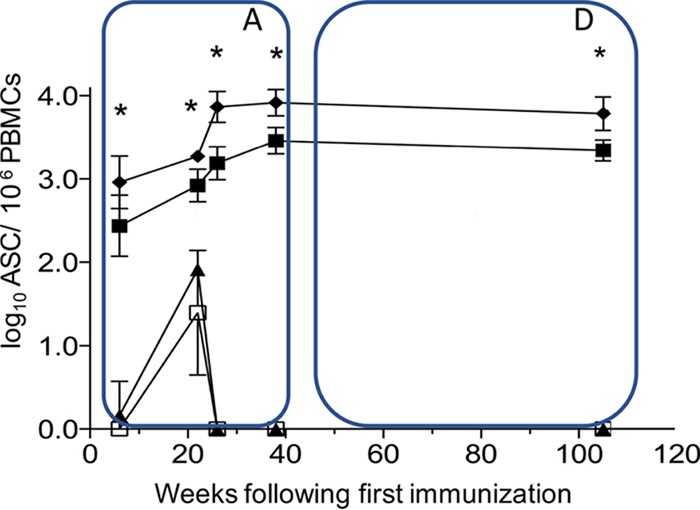
Env-specific B cells in peripheral blood (ASC/1 × 10^6^ PBMC) per group during the immunization phase (box A) (at 0, 4, 24, and 36 weeks) and after 70 weeks of long-term follow-up (box D). The frequency of Env-specific memory B cells was determined by B-cell ELISpot assays in animals immunized with gp140 CD4bs-occluded group 1 (black diamonds) or gp140 CD4bs-open group 2 (black squares). Control groups included group 3 (mini-CD4 mimetic only, lower value; black triangle) and group 4 (MF59 only; open squares). Immunizations were given at weeks 0, 4, 24, and 36 (box A). The values (numbers of ASC per 10^6^ PBMCs) are means from 6 animals per group ± standard deviations (error bars). *, *P* > 0.001 (two-way ANOVA).

Throughout the immunization phase (Fig. 4, phase A) of the study, the gp140CD4bs-x (CD4bs-occluded) vaccine group developed significantly (*P* < 0.001) higher numbers of Ag-specific B cells than the gp140 (CD4bs-open) group. Despite the expected individual variation in the number of circulating Env-specific B-cell immunosorbent spots found, there were consistent and significant differences between the two Env-vaccinated groups. Two weeks after the second immunization, these numbers were highest in group 1 (gp140CD4bs-x), ranging between 250 and 1,865 immunosorbent spots/10^6^ peripheral blood mononuclear cells (PBMC) versus a lower range, between 105 and 975 immunosorbent spots/10^6^ PBMCs, for macaques immunized with gp140 alone. Both groups reached statistical significance, with the level for the gp140CD4bs-occluded group being consistently higher by the second immunization and increasing further after the third immunization, reaching a peak plateau in the gp140CD4bs-occluded group with a range from 3,630 to 11,823 immunosorbent spots/10^6^ PBMCs by week 26 (Fig. 4, phase A). Macaques receiving gp140 (CD4bs-open) required a fourth immunization to reach a plateau, and the responses were at a lower range, from 1,550 to 4,365 immunosorbent spots/10^6^ PBMCs by week 38 (Fig. 4, phase A). The CD4bs-occluded group had significantly (*P* < 0.001) and consistently higher numbers of circulating Ag-specific B cells than the CD4bs-open group throughout the immunization schedule. This demonstrated a significant and positive impact on the kinetics of priming and development of B-cell responses by simply altering the CD4bs on Env antigen to prevent CD4 receptor binding by Env vaccine antigen. A minor background response in the CD4 mimetic minipeptide and adjuvant control groups was detectable at only one time point (week 20) over the entire study.

### Antibody titers to total Env are primed first and peak earliest in CD4bs-occluded Env-immunized animals.

**FIG 5:**
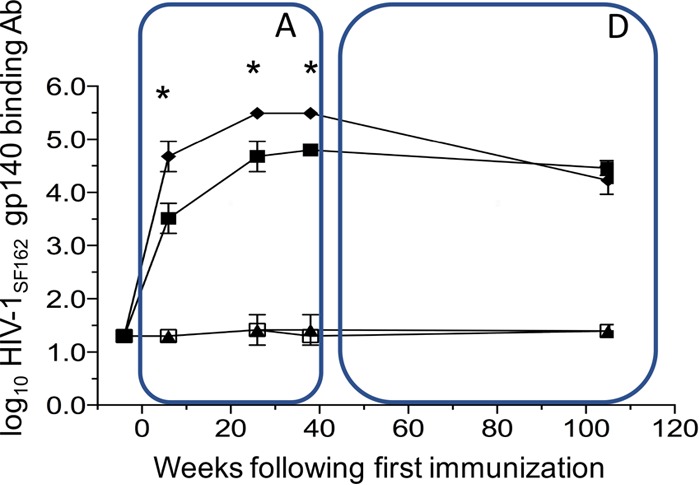
Kinetics of anti-Env titers per group during the immunization phase (at 0, 4, 24, and 36 weeks) (box A) and after 70 weeks of long-term follow-up (box D). HIV-1_SF162_ gp140-specific binding antibody responses induced after immunization with gp140 CD4bs-occluded (group 1; black diamonds), gp140 CD4bs open (group 2; black squares), group 3 immunization mini-CD4 mimetic (black triangles), and control group 4, immunized with MF59 only (open squares). The values (binding endpoint titers) are means from 6 animals per group ± standard deviations (error bars). *, *P* > 0.0001 (two-way ANOVA).

During the active immunization phase of the study, the early appearance of antibodies reflected the early appearance of Ag-specific B cells in circulation in the CD4bs-occluded Env group compared to the CD4bs-open gp140 Ag (Fig. 5, phase A). Levels of total Env binding antibodies were detectable and higher within 6 weeks, 2 weeks after the second immunization in the CD4bs-occluded Env-immunized group, and they were already comparable to mean Env titers observed in the gp140 group after the third immunization (Fig. 5, phase A). Titers again increased markedly after the third immunization, peaking at week 24 and reaching a plateau at week 40. Antibody titers in these immunized macaques were significantly (*P* > 0.0001 by two-way analysis of variance [ANOVA]) greater than those induced with gp140 (CD4bs-open) during the active immunization phase (A), 4 weeks after the fourth immunization, suggesting a positive impact of occluding the CD4bs of gp140 on priming and the magnitude of anti-Env titers reached during the immediate immunization period (Fig. 5, phase A).

### Neutralizing antibodies peak earlier and show activity against HIV-2 in the presence of soluble CD4 (sCD4).

To accurately assess neutralization of HIV-1 enveloped viruses in rhesus macaques, we turned to *in vivo* rhesus-adapted simian-human immunodeficiency viruses (SHIV) using the lentivirus pseudotype system to avoid differences in non-enveloped-encoded differences in their genomes.

**FIG 6:**
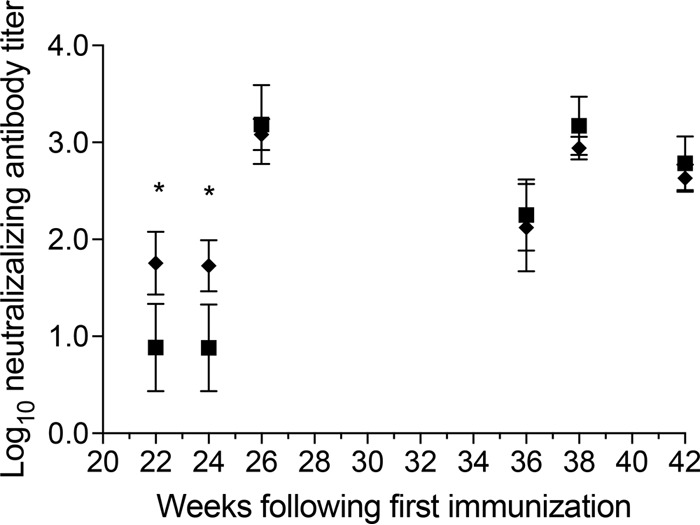
Early induction of neutralization responses in gp140 CD4bs-occluded (black diamonds) and gp140 CD4bs-open immunized animals (black squares). Neutralization of relatively homologous SHIV_SF162p4_ by sera from immunized animals is shown. Antibody titers are expressed as the dilution of serum required to reduce the luciferase activity in cultures exposed to SHIV_SF162p4_ pseudovirus alone by 50%. The values are means from 6 animals per group ± standard deviations (error bars). Elevated early responses were detected 18 and 20 weeks after the second immunization (administered at 4 weeks) and 22 and 24 weeks after the first immunization. Subsequently, homologous neutralization titers became similar in both Env immunized groups. *, *P* < 0.001 (one-way ANOVA).

Importantly, early in the immunization protocol, CD4bs-blocked gp140-immunized animals induced the first neutralizing antibody response to the relatively homologous tier 1 SHIV_SF162P4_, observed at weeks 22 and 24 (*P* < 0.001 by one-way ANOVA) (Fig. 6). Subsequently, neutralizing antibody titers increased after each immunization until the third immunization, when slower-developing neutralizing titers in the gp140 group had eventually caught up with the CD4bs-blocked group. By the third immunization (week 24), there was a boost in homologous neutralizing titers in the gp140 group that reached levels similar to those of the CD4bs-blocked group (Fig. 6). Given that globally the Env-neutralizing epitopes were otherwise identical in the CD4bs-blocked versus CD4bs-open gp140, this was not unexpected (with the exception of fine specificity differences of CD4i epitopes caused by CD4bs-M48U1 cross-linking [27]).

Due to the slower acquisition of heterologous neutralizing antibodies, sera collected at weeks 38 and 42 (2 or 6 weeks after the 4th immunization) were measured for neutralization against clade B SHIV virus strains. In the pseudotype system, these included tier 1 SHIV_89.6_, SHIV_W6.1D_, tier 2 SHIV_SF162p3_, and HIV-2.

**FIG 7:**
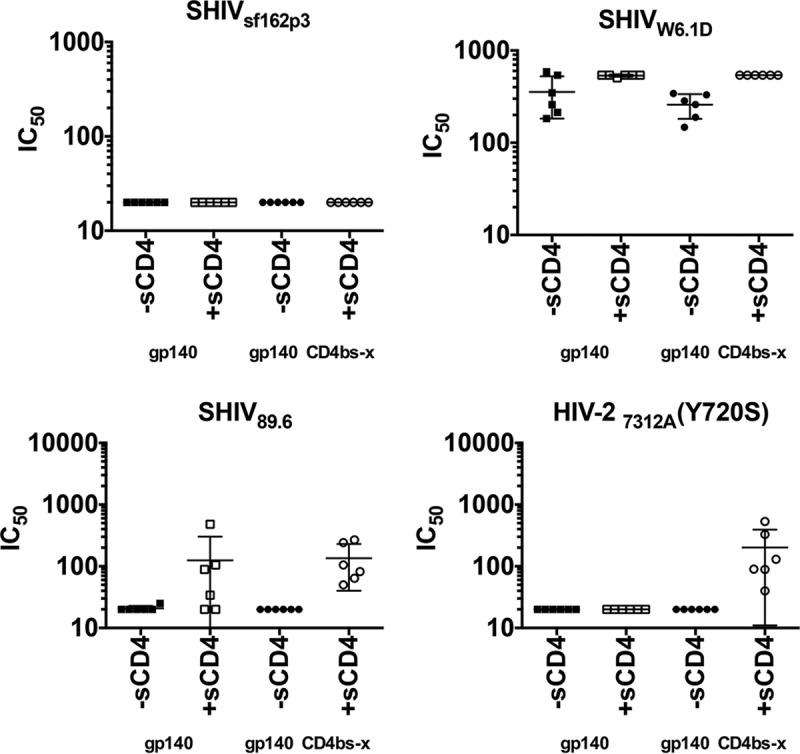
Heterologous neutralizing activity in the presence or absence of soluble CD4 during the immunization phase. Heterologous neutralization of a panel of clade B SHIV pseudoviruses with sera taken at 2 or 6 weeks after the 4th vaccination is shown. Comparison of neutralization activity of sera from animals immunized with gp140-CD4bs-x (occluded group; circles) in the absence (−sCD4) or presence of sCD4 (+sCD4) versus gp140 CD4bs-open (squares) also is shown. To confirm that the CD4i epitopes in the gp140-CD4bs-x immunized group (1) were exposed and functional, sera were tested against the HIV-2_7312A_ pseudovirus. Fifty percent inhibitory concentrations (IC_50_) against different viral isolates are indicated. The symbols represent values from individual animals, while the horizontal bars are means from 6 animals per group ± standard deviations (error bars).

HIV-1 gp120 bound to CD4 gives a stable conformation that presents an increased affinity for the chemokine receptors and CD4i antibodies that also broadly neutralize HIV-2 (28–31). We used sCD4 in our assays to determine if CD4-inducible neutralizing antibody responses remained intact in the CD4bs-occluded gp140 group (32). The mean 50% neutralization titers against tier 2 SHIV_SF162p3_ were <20 (in the presence or absence of sCD4) (Fig. 7). Against SHIV_89.6_, the mean titers were undetectable (without sCD4) and 1:125 (with sCD4), while against the tier 1 SHIV_W6.1D_ the mean titer was 1:355 (without sCD4) and even higher (with sCD4) in the gp140-immunized group. The mean titers in the CD4bs-blocked gp140-immunized group were <20 (both against the tier 2 SF162p3 and 89.6 pseudoviruses) and 1:259 against the tier 1 W6.1D virus, all without sCD4, while the mean titers in the presence of sCD4 were <20, 1:135, and higher than 1:540, respectively. Of note, this gp140 was chosen for this proof-of-concept CD4bs-occlusion study because the protein was well characterized and used in many previous studies, not because of the bNAb epitopes it presented. It was also selected because of its neutralization inducing potential for CD4i, one of the control features for the M48U1 occlusion. Importantly, we observed neutralizing responses against HIV-2 (Y720S) in the presence of sCD4 in the CD4-bound gp140 group, confirming that the CD4i epitopes were exposed, functional, and induced by this immunogen (Fig. 7), and this was independently confirmed by Shen et al. (27).

### Superior ADCC responses elicited by immunization with CD4bs-occluded Env immunogen.

**FIG 8:**
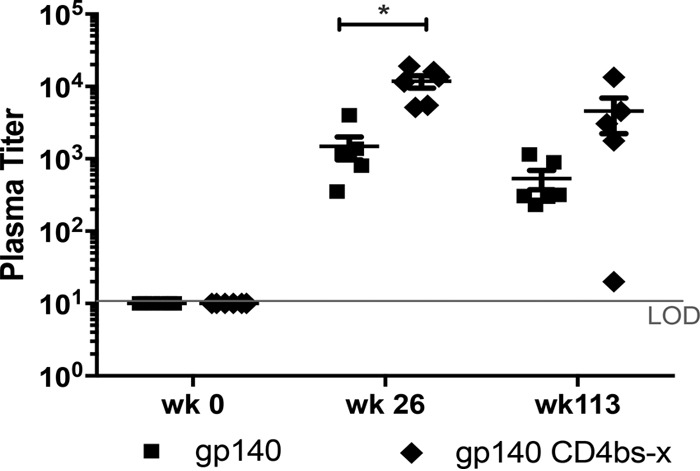
ADCC activity in CD4bs-x (occluded; black diamonds) Env-immunized animals versus CD4bs-open (black squares) Env-immunized animals. Data shown are 2 weeks after the third immunization and after long-term follow-up after the 4th immunization (*, *P* = 0.014). LOD, limit of detection.

Nonneutralizing antibodies are becoming recognized as important vaccine-induced effector responses in protective HIV-1 immunity. Antibody-dependent cellular cytotoxicity (ADCC) responses have been correlated with slower disease progression (33–35) as well as vaccine efficacy (36–38). A high proportion of ADCC responses in patient sera are directed toward CD4i epitopes (39), the same epitopes we have preserved by stabilizing them with our CD4bs-linked CD4 mimetic complex, as we previously validated (22, 40). To determine if ADCC activity was induced by Env immunization with the CD4bs-occluded or CD4bs-open gp140, sera from immunized animals were assayed at weeks 0 and 26 and after long-term follow-up. ADCC activity was measured as the serum titer for mediating granzyme B (GzB) release by PBMC upon incubation with target cells coated with SF162 gp120 protein. Group 1, immunized with gp140 CD4bs-occluded, had statistically superior ADCC responses compared to group 2, immunized with gp140 with CD4bs-open, with a false discovery rate (FDR) *P* value of 0.014 (Wilcoxon rank-sum exact test; *P* values were controlled for FDR with the Benjamini-Hochberg method) at week 26 (Fig. 8).

### Circulating antigen-specific IL-4 CD4 T-cell responses increase early during immunization with CD4bs-occluded Env immunogen.

**FIG 9:**
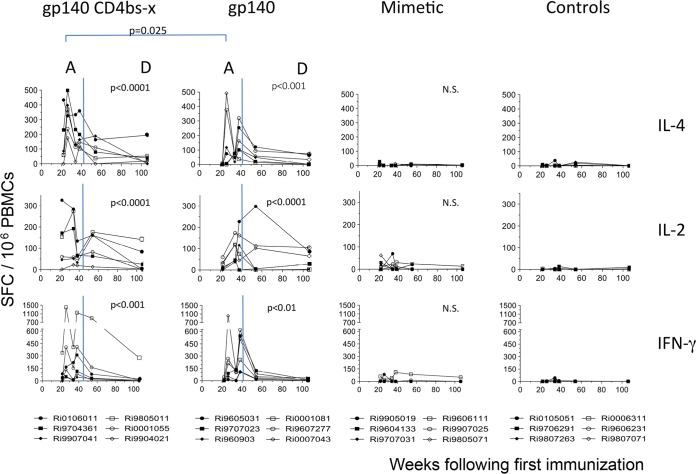
Env-specific cytokine-secreting T-cell immunosorbent spot assay responses following immunizations at 0, 4, 24, and 36 weeks (immunization phase) (A) and long-term 70 weeks follow-up (durability phase) (D). Shown are IL-4 (upper row), IL-2 (middle row), and IFN-γ (lower row) secreting spot-forming cells over time. Immunosorbent spot assays from individual animals immunized with gp140-CD4bs-x (occluded group; first column), gp140-CD4bs-open (second column), mini-CD4 mimetic only (third column), and adjuvant only (fourth column). Background responses (mean numbers of spots plus 2× the standard deviations from triplicate assays with medium alone) were subtracted. Responses after stimulation with overlapping SF162 gp120 20mer peptides are presented as the number of spot-forming cells per 10^6^ PBMCs. N.S., no significance.

Immunization with both HIV-1 Env immunogens induced T-cell immunosorbent spots specific for peptides of the external HIV-1 envelope glycoprotein (gp120) that were detectable during the active phase of immunization (Fig. 9, phase A) but which tapered off during the long-term follow-up period (Fig. 9, phase D). These increases were observed with lymphocytes producing IL-4, IL-2, or IFN-γ and reached statistical significance relative to the 2 groups of control macaques (mimetic and adjuvant controls). These data were analyzed in a two-way analysis of variance with all time points included where data from all 24 macaques were available.

Importantly, the earliest and most robust T-helper responses were observed in macaques immunized with gp140-CD4bs-occluded, which induced the highest number of IL-4-producing immunosorbent spots found at week 26 (*P* = 0.025) (Fig. 9, phase A) for both gp120 and gp41 sets of peptides (HIV-1 gp120, *P* < 0.0001 [Fig. 9, phase A]; HIV-1 gp41, *P* < 0.0001 [gp41 data not shown]). The highest number of IL-2-producing immunosorbent spots recognizing HIV-1 gp120 peptides was seen after three immunizations at week 34 (*P* < 0.01). The IFN-γ immunosorbent spots peaked at week 38 (gp120, *P* < 0.0001) (Fig. 9, phase A). All six gp140-immunized macaques induced more than 50 IFN-γ-producing lymphocytes per million PBMCs at week 38, while five of the macaques immunized with the CD4bs-occluded Ag induced this same level of ELISpot activity and four had more than 50 IL-2- and IL-4-producing lymphocytes. While macaques immunized with gp140 only did not have increased numbers of HIV-1 gp41-specific, IFN-γ-producing immunosorbent spots overall, numbers were significantly increased, relative to control macaques, at week 38.

Immunizations at weeks 24 and 36 each produced an increase in the number of T-cell immunosorbent spots recognizing either HIV-1 gp120 or gp41 peptides in both the gp140-alone and gp140 CD4bs-occluded groups (Fig. 9, phase A, and data not shown). This pattern was observed following each immunization, with the exception of IL-2 immunosorbent spots at week 36. Also with the exception of IL-2 at week 38, immunosorbent spot numbers at weeks 26 and 38 were statistically significantly higher than those of controls at these time points. Peak levels were found after four immunizations (week 38; γ-IFN, *P* < 0.0001; IL-4, *P* < 0.0001). The gp140-CD4bs-occluded and gp140-alone groups induced statistically significant numbers of IL-4-producing, but not IL-2-producing, immunosorbent spots recognizing peptides from the HIV-1 transmembrane envelope glycoprotein (gp41) (data not shown). The numbers of immunosorbent spots responding to gp41 peptides were smaller and delayed, with the highest levels seen after five immunizations for all three cytokines (data not shown). In summary, during the active immunization phase (Fig. 9, phase A), CD4bs-occluded gp140 immunization induced earlier and more robust IL-4 (week 26; *P* = 0.025), as well as a trend for earlier IL-2 responses (*P* = 0.065), suggesting a more vigorous and early recruitment of CD4^+^ T-helper cells during the inductive phase of the B-cell response (Fig. 4, phase A).

### Durability of B-cell responses.

A key concern of HIV-1 vaccine development has been the very poor durability of HIV-1 vaccine-induced responses, especially B-cell responses (38, 41–43). To address this, after an active immunization phase with 4 immunizations given over 40 weeks, we embarked on a long-term follow-up phase where animals were rested without sedation and protocol bleeds for approximately 70 weeks (1.5 years) to determine the durability of B- and T-cell immune responses.

Importantly, while the plasma antibody titers gradually contracted after boosting, the superior numbers of Ag-specific B cells in circulation were sustained in the CD4bs-occluded group throughout the long-term follow-up with a slight decline over the long 70-week period (Fig. 4, phase D). Despite these impressive and durable levels of Ag-specific B cells during the active phase (Fig. 4, phase A) and the early higher titers and peak of total anti-Env antibodies in the CD4bs-occluded group, during the 70 weeks of observation in the durability phase (Fig. 5, phase D), total Env titers began to wane. The higher titer of total Env antibodies slowly decayed, and after the 1-year endpoint (week 107) the levels were similar to those of the gp140-open-immunized animals (Fig. 5 phase D). This suggested that the global antibody response produced by the plasma cell pool that had accumulated during immunization had reached a maximum equilibrium despite the impressive and sustained kinetics of the Ag-specific memory B cells in circulation.

Control macaques immunized with the CD4 mimetic or adjuvant alone had no specific antibody to gp140. Macaques immunized with gp140 CD4bs-open produced low levels of antibodies that transiently cross-reacted with the CD4-mimetic peptide at week 26 (data not shown). Antibodies to the mimetic peptide were not detectable in other macaques.

Remarkably, the long-term durability of Env-specific memory B cells in circulation correlated with more robust ADCC responses (*P* < 0.001; Spearman's coefficient, 0.85), which were also found to be durable during the extended long-term follow-up of more than 70 weeks (Fig. 8).

## DISCUSSION

This study set out to determine if rational HIV-1 Env-antigen design could improve B-cell responses in primates. High-affinity CD4 binding associated with HIV-1 Env residue 375 substitutions has been associated with increased virulence in macaques (44). We reasoned that if we could prevent CD4 binding by envelope antigens yet preserve key CD4-inducible (CD4i) epitopes, which were important for virus neutralization and rich in ADCC epitopes, then we could provide the basis for an improved HIV-Env antigen scaffold which could be suitably modified for future presentation of key broadly neutralizing epitopes and, ultimately, the deletion of dominant nonconserved antigen decoys. Our design criteria were 2-fold, namely, CD4 binding site occlusion and preservation of CD4i NAb epitopes, both of which we have demonstrated in previous structural and small-animal studies (22, 25, 26). Our immunological criteria required an *in vivo* primate CD4 T-cell system compatible with HIV-1 Env binding to study the *in vivo* inductive events in the presence of functional CD4 gp140 interaction *in vivo*. Our immunological criteria included improved B-cell responses with respect to magnitude and durability, preservation of CD4i epitopes and induction of ADCC, and evidence for improvement of one or more of the antigen-specific T-cell subsets (IFN-γ, IL-2, and IL-4).

When HIV-1 gp120 binds to CD4, it stabilizes the virus envelope in a conformation that presents an increased affinity for the chemokine receptors and CD4i antibodies (28–31). Therefore, the bound envelope glycoproteins offer different targets both to induce and bind antibodies. Recent studies evaluating the evolution and specificities of broadly neutralizing antibodies during HIV-1 infection (32, 45, 46) have provided important insights regarding the significance of CD4i antibodies and their potential role in vaccines against HIV-1. So far, recombinant monomeric gp120 or oligomeric/trimeric gp140 glycoproteins have failed to elicit broad and potent neutralizing antibodies in experimental animal models. Past studies based on gp120-CD4 (or CD4 mimic) complexes or constrained core gp120 antigens have been evaluated as vaccine candidates, aiming at inducing CD4i antibodies (26, 47–50). Fouts et al. demonstrated that gp120 cross-linked to CD4 D1D2 domains raised antibodies that neutralized primary viruses regardless of coreceptor usage and genetic subtype in nonhuman primates (48). These findings were extended in a challenge study by DeVico et al. (47), where macaques immunized with a single-chain complex containing gp120BaL-rhesus macaque CD4 D1D2 showed improved CD4i antibody responses that correlated with the control of infection when challenged with SHIV_SF162P3_. Although this correlation did not prove that efficacy was mediated by neutralizing CD4i antibodies, it demonstrated that the presence of CD4i Abs was dependent on the CD4-bound conformation of HIV-1 envelope *in vivo*. These studies demonstrated the potential importance of strategies directed to raising antibodies against the CD4i site. Recently we used the practical approach of eliciting CD4i epitope-directed virus-neutralizing antibodies using a stably cross-linked complex of recombinant oligomeric gp140 and CD4 mimetic miniproteins (mini-CD4) (M64U1-SH) (22, 51) to target the conserved coreceptor binding site of the HIV-1 Env. In those studies, two mini-CD4 were cross-linked to various forms of HIV-1 Env (M64U1-SH). Based on results from those studies, the M64U1-SH mini-CD4 was selected for generating the cross-linked gp140–mini-CD4 complex.

Two important and underappreciated issues were addressed in this study. First was the observation that binding or cross-linking of the CD4 molecule of T-helper cells causes functional impairment within germinal centers (11, 12, 52–54). The hypothesis was that CD4 binding by antigen would impair critical interactions between Ag-specific CD4 Tfh cells and MHC-II B cells, which are fundamentally important in generating memory B-cell responses, and functionally important antibody effector responses, such as NAb and ADCC. The second was that CD4i epitope regions are also rich in ADCC epitopes (39); thus, stabilizing their presentation would promote such ADCC in naive vaccinated individuals. Importantly, in this study, by simple cross-linking of the small CD4 receptor mimetic to the CD4 binding site of gp140, we have been able to demonstrate (i) preservation of CD4i NAb, (ii) improved and long-term durable B-cell responses, (iii) early induction of anti-HIV-1 binding and NAbs, and (iv) ADCC. The early and robust CD4 T-helper responses characterized by IL-4 secretion correlated with the early induction of B-cell and antibody responses, suggesting that preventing CD4 binding of the Env antigen in B-cell-inductive sites was an underlying and important feature of this antigen modification. These findings beg mechanistic follow-up studies to prove this hypothesis and to understand the *in vivo* half-life and kinetics of gp140 in the CD4bs-occluded, M48U1-complexed versus the unbound forms in lymph nodes draining vaccine injection sites. Most notably, the induced B-cell responses were durable for more than 1.5 years postimmunization, representing a major advance in a key area of HIV vaccine development. Future modifications to further improve Env antigen structures with additional modifications to better present and recruit key bNAb and ADCC epitope-rich regions are likely to ultimately contribute to more highly effective HIV-1 vaccines.

## MATERIALS AND METHODS

As the well-characterized model of HIV-1 immunogen, which has been used in human and nonhuman vaccine trials, we used the recombinant HIV-1 gp140 of the subclade B SF162 (55, 56), from which pathogenic SHIV was developed (57). To prevent CD4 receptor engagement, a mini-CD4 peptide (M64U1-SH) was used to cross-link gp140dV2SF140, as described by Dereuddre-Bosquet et al. and Van Herrewege et al. (51, 58). Cross-linking of the minipeptide mimicking the CD4 receptor binding site to gp140 was described by Martin et al. (22), and it effectively (Fig. 1) (23) occludes critical Env CD4bs sites, such as residue 375 (44).

### Animals and immunizations.

A total of 24 mature captive-bred male rhesus macaques (Macaca mulatta) were housed at the Biomedical Primate Research Centre (BPRC), The Netherlands. The rhesus macaques were negative for antibodies to SIV, simian type D retrovirus, and simian T-cell lymphotropic virus at the initiation of the study. The study protocol and experimental procedures were approved by the institute's animal ethical care and use committee and were performed in accordance with Dutch law and international ethical and scientific standards and guidelines. Behavior, discomfort, and appetite were observed daily during the study by specially trained personnel. Body weight and body temperature were measured before the start of the experiment and each time the animals were sedated for immunization and/or blood sampling immediately following sedation of each individual animal.

The study consisted of 24 animals divided into four groups of 6, randomized based on age and weight. All groups received 0.5 ml of the MF59 adjuvant intramuscularly (i.m.) (upper leg) to formulate the protein, while the last group served as the adjuvant-only control. Group 1 animals were immunized with gp140 with its CD4bs blocked (gp140 CD4bs-x; 100 μg of gp140_ΔV2SF162_ with the CD4bs blocked by the mimetic M64U1-SH). Group 2 animals were immunized with the same but unblocked gp140 (100 μg of gp140_ΔV2SF162_), group 3 received the mimetic M64U1 as a control, and group 4 was the adjuvant-only control group (0.5 ml of the adjuvant MF59). All animals were immunized at weeks 0, 4, 24, and 36. To assess the durability of vaccine-induced responses in animals, immune responses were monitored up to week 107 (71 weeks after the 4th immunization) (Fig. 3).

For immune assays, serum, plasma, and PBMC were isolated from blood samples collected from sedated animals (ketamine hydrochloride anesthesia at 10 mg/kg of body weight) at regular time intervals aseptically (Vacutainer; Becton Dickinson). To investigate possible adverse effects, body weight, rectal temperature, routine hematology, and clinical chemistry were monitored at regular intervals.

### Humoral immune responses: binding antibody titers and neutralization assay.

Antibodies to HIV-1 SF162 Env (gp140) in serum were measured by enzyme-linked immunosorbent assay (ELISA). Plates were coated overnight with Env in 100 mM NaHCO_3_ and were blocked for 1 h with 1% nonfat milk before application of serum serially diluted in 1% bovine serum albumin (BSA)-phosphate-buffered saline (PBS) buffer. After 1 h, 1 μg/ml anti-human IgG-horseradish peroxidase (HRP) conjugate was added for an additional hour before the addition of ultra-TMB ELISA development reagent. The reaction was stopped by addition of 0.5 M H_2_SO_4_. Results were expressed as IgG endpoint dilution titers.

For the standardized and validated neutralization assays, the TZM-bl cell line was used (59, 60). It was obtained through the NIH AIDS Research and Reference Reagent Program, Division of AIDS, NIAID, NIH, from John C. Kappes, Xiaoyun Wu, and Tranzyme, Inc. The HeLa cell line was engineered to express CD4 and CCR5 receptors. Following infection with SHIV-pseudotyped virus, the cells produce luciferase, the activity of which was detected by chemiluminescence. Sera were diluted to give a 1:20 dilution and subsequently in a 3-fold series to a final dilution of 1:43,740. Each dilution was mixed with sufficient pseudovirus to give 500,000 cps in a Perkin-Elmer Victor 6016971 luminometer. The mixture included 15 μg/ml of DEAE and was incubated for 1 h before 10,000 TZM-bl cells were added. The cells were cultured for 48 h, the supernatants were removed, and the cells were lysed. The cell lysates were transferred to black/white plates, britelite reagent was added, and the luciferase activity was quantified. Antibody titers are expressed as the dilution of serum required to reduce the luciferase activity in cultures exposed to pseudovirus alone by 50% (61–63). As a positive control for the detection of CD4i neutralizing antibodies, a modified neutralization assay using HIV-2_7312A_ pseudovirus was used as previously described (32).

### B-cell ELISpot assays.

Antigen-specific B-cell counts were performed as described by Crotty et al. (64). PBMC were plated in 48-well plates at 1 × 10^6^ cells/ml in complete medium (RPMI 1640 with l-glutamine, penicillin-streptomycin, HEPES buffer, and 10% fetal bovine serum) containing PWM (pokeweed mitogen) at a dilution of 1:10,000; SAC (Staphylococcus aureus Cowan strain 1) at a dilution of 1:10,000; β-mercaptoethanol at a dilution of 1:1,000; 20 U/ml IL-2, IL-4, IL-5, and IL-6; and CpG oligonucleotide at a concentration of 5 μg/ml. Plates were incubated at 37°C, 5% CO_2_, for 5 days. To enumerate Ag-specific B-cell antibody-secreting cells (ASC) or spot-forming units (SFU), 96-well plates were coated with 50 μl/well gp120 SF162 Env antigen at a final concentration of 5 μg/ml. After 18 h, plates were washed and blocked with 100 μl/well complete medium at 37°C for 1 to 2 h prior to use.

On day 6, the cells were washed thoroughly, plated onto the ELISpot plates, and incubated at 37°C and 5% CO_2_ overnight. Plates were washed with PBS followed by PBS containing 0.05% Tween 20 (PBST). Plates then were incubated overnight in 1 μg/ml biotinylated goat-anti-rhesus Ig (Hybridoma Reagent Laboratory) in PBST with 2% fetal calf serum (FCS). Plates were washed again, developed using 5 μg/ml HRP-conjugated avidin diluted in PBST, and incubated for 1 to 2 h at 37°C. Plates were washed again and then developed using 3-amino-9 ethyl-carbazole (AEC; Sigma), giving spot formation. The reaction was stopped by washing the plates with tap water. Spots were counted using the A.EL.VIS ELISpot reader. Data are presented as ASC per 1 × 10^6^ PBMC.

### ADCC assays.

ADCC assays were performed, as previously described by Pollara et al. (65), using CEM.NKR_CCR5_ cells coated with recombinant HIV-1 gp120 SF162 as target cells and PBMC obtained from an HIV-seronegative donor as effector cells. The ADCC-mediating antibody titer was defined as the reciprocal of the highest dilution indicating a positive GzB response (>8% GzB activity) after background subtraction, as previously described (65).

### T-cell ELISpot assays.

Enumeration of antigen-specific IFN-γ, IL-2, and IL-4 cytokines was measured using an ELISpot assay as described by Koopman et al. (66). Separate peptide pools, consisting of 15mers with an 11-amino-acid overlap, which covered the entire gp41 and gp120 of SF162 (NIH AIDS Reagent Program), were used to measure antigen-specific immune responses after each immunization, during follow-up, and after challenge. Medium alone was used as a negative control, while stimulation with phorbol myristate acetate (20 ng/ml) plus ionomycin (1 μg/ml) was used as a positive control. In brief, 4 × 10^6^ cells/ml were stimulated in RPMI 1640 medium supplemented with 10% FCS in a 24-well tissue culture plate for 24 h. For the enumeration of antigen-specific cytokine production, nonadherent cells were collected and plated at 2 × 10^5^ cells/well in triplicate in a 96-well ELISpot plate with the same antigen. Microtiter plates were precoated with the following monoclonal antibodies (MAbs): anti-IFN-γ MAb (MD-1; U-Cytech, Utrecht, The Netherlands), anti-IL-4 MAb (QS-4; U-Cytech), and anti-IL-2 MAb (B-G5; Diaclone Laboratories, Besançon Cedex, France). Detection of the cytokine-secreting cells took place after 15 h for IL-4 and 4 h for IFN-γ and IL-2. The cells were lysed and the debris was washed away before adding detector antibodies. IFN-γ, IL-2, and IL-4 were detected using biotinylated rabbit-anti-rhesus IL-2, biotinylated rabbit-anti-rhesus IFN-γ, or biotinylated mouse-anti-rhesus IL-4 (U-Cytech). Spots were visualized using streptavidin-HRP and an AEC coloring system.

### CD4 T-cell proliferation inhibition assay.

PBMC were incubated overnight at 4°C in RPMI with 10% FCS containing SF162 gp140 (CD4bs-open) (1 μg/ml/10^6^ cells), SF162 gp140CD4bs-occluded (1 μg/ml/10^6^ cells), or no additions. Cells were subsequently harvested and labeled with CellTrace (20 min of incubation at 37°C, 1 μl CellTrace/ml/10^6^ cells; CellTrace violet cell proliferation kit; Molecular Probes, Invitrogen, Carlsbad, CA, USA). Cells then were incubated for 72 h on CD3-coated microwell plates (coated with 1 μg/ml CD3 clone SP34 [Becton & Dickinson] with 2 h of incubation at 37°C and then 3 washes with PBS) either without additions, with SF162 gp140 (CD4bs-open) (1 μg/ml), or with SF162 gp140CD4bs-occluded (1 μg/ml). Cells were then stained with CD3^APC^ and CD4^PE-Cy7^, and expression of CellTrace label was detected by FACS analysis.

### Statistical analyses.

The statistical significance of differences between responses induced by the different (CD4bs-occluded or CD4bs-open gp140) immunogens was determined by Dunnett's multiple-comparison test (one-way ANOVA), Dunn's multiple-comparison test (nonparametric test), or Bonferroni posttests (two-way ANOVA). For IFN-γ and IL-4, the area under the curve (AUC) was calculated for weeks 22 to 26, whereas week 22 data were used for IL-2 (week 26 data were not available). Correlations between cytokine AUC, B-cell ELISpot assay result, and ADCC were then assessed using Spearman's rho (nonparametric test). Immunosorbent spot assay AUC were calculated for the induction phase (weeks 22 to 38), and CD4bs-occluded- or CD4bs-open gp140-immunized groups were compared nonparametrically (Mann-Whitney U test).
